# Regulatory Roles of Kaempferol on the PI3K/AKT/mTOR Signaling Pathway and Associated MicroRNAs in Different Cancer Types

**DOI:** 10.3390/ijms27167420

**Published:** 2026-08-19

**Authors:** Önder Yumrutaş, Miguel Rios, Pınar Yumrutaş, Murat Korkmaz, Jorge Escobar, Ali Parlar, Jose L. Martínez

**Affiliations:** 1 Department of Medical Biology, Faculty of Medicine, University of Adıyaman, Adıyaman 02200, Turkey; 2 Department of Biology, Faculty of Chemistry and Biology, Universidad de Santiago de Chile, Santiago 9170022, Chile; 3 Department of Pharmacology, Faculty of Medicine, Adıyaman University, Adıyaman 02200, Turkey; 4 Department of Medical Biology, Faculty of Medicine, Gaziantep Islam Science and Technology University, Gaziantep 27010, Turkey; 5 Biological Chemistry Laboratory, Faculty of Sciences, Pontifical Catholic University of Valparaíso, Valparaíso 2340000, Chile; 6 Department of Metallurgical Engineering, Faculty of Engineering, Universidad de Santiago de Chile, Santiago 9170022, Chile; joseluis.martinez@usach.cl

**Keywords:** kaempferol, miRNA, cancer, PI3K/AKT/mTOR, anticancer, bioactivity

## Abstract

Kaempferol, a naturally occurring dietary flavonoid abundantly found in various fruits and vegetables, has garnered significant attention due to its diverse pharmacological properties, most notably its potent anticancer activity. In the orchestration of cancer pathogenesis, the PTEN/PI3K/AKT/mTOR signaling cascade plays a pivotal role, where aberrant activation or dysregulation of this pathway drastically accelerates tumor cell proliferation, survival, and metabolic reprogramming, thereby driving tumorigenesis. Moreover, non-coding microRNAs (miRNAs) have emerged as key modulators involved in regulating this pathway, functioning as either oncogenes or tumor suppressors to determine cancer cell fate. Previous studies have demonstrated that kaempferol and its derivatives, owing to their molecular structures, suppress the PI3K/AKT/mTOR signaling pathway either directly or indirectly, thereby playing a critical role in inhibiting cancer cell proliferation. Hence, this comprehensive review elucidates the therapeutic potential of kaempferol and its derivatives, focusing specifically on their molecular mechanisms in modulating the PTEN/PI3K/AKT/mTOR pathway. Furthermore, this review highlights the crosstalk between kaempferol and the specific miRNAs targeting these signaling components in various cancer types.

## 1. Introduction

**Figure 1:**
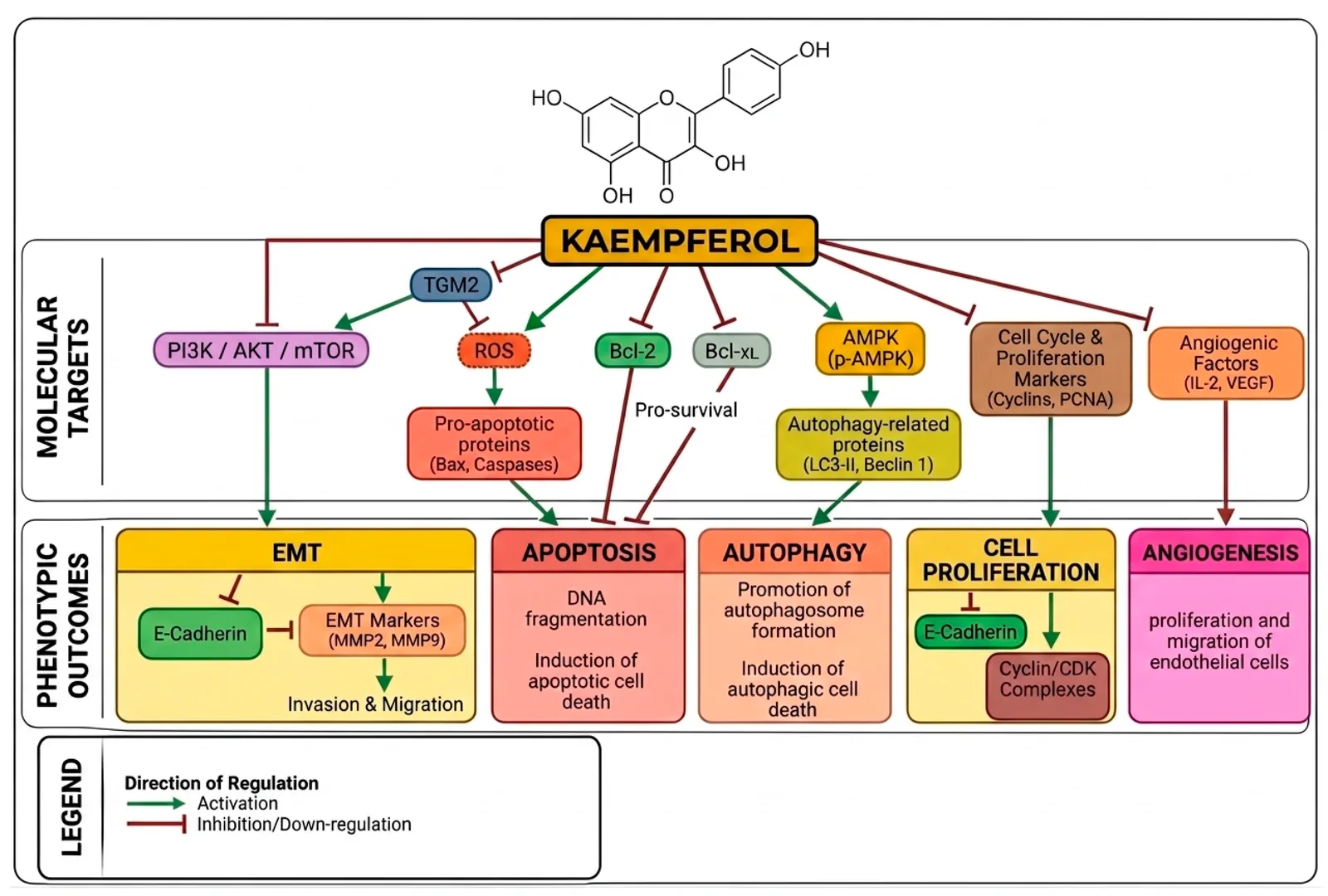
Molecular mechanisms and signaling pathways regulated by kaempferol in cancer cells.

Historically, plants have served as vital natural resources for both nutritional and medicinal applications. In addition to their direct use as spices, teas, and food, determining the bioactivities of phytochemicals derived from these plants plays a crucial role. Among the isolated phytochemicals, phenolic compounds contain subgroups such as flavonoids, anthocyanins, tannins, and phenolic acids. These compounds possess significant biological activities, and numerous studies aiming to determine these activities have been reported in the literature [1,2]. The flavonoids from these subgroups have been identified in various plant parts, including fruits, vegetables, bark, roots and flowers [3]. They are subdivided into flavones, flavonols, flavanones and isoflavonoids, neoflavonoids, flavanols, anthocyanins, and chalcones. These compounds exhibit various bioactivities, including antioxidant [4,5], antiviral [6], anticancer [7], anti-inflammatory [5,8], and protection from cardiovascular injury [9], making them indispensable across the pharmaceutical, medical, and cosmetic industries [3]. Moreover, it is thought that flavonoids may be clinically useful in the treatment of many diseases, especially cancer and cardiovascular disorders [10,11]. The most well-known flavonoids are generally kaempferol, luteol, genistein, epicatechin, rutin, quercetin, and apigenin. It has been proven in different studies that these compounds exhibit significant bioactivities due to their chemical structure. Among these compounds, kaempferol (3,5,7-trihydroxy-2-(4-hydroxyphenyl)-4H-chromen-4-one) has been shown to possess a broad spectrum of biological activities (Figure 1) [2,12,13]. In particular, kaempferol exhibits powerful anticancer activities in various cancer cells such as breast [14,15], ovarian [16], colon [17,18], gastric [19], lung [20], pancreatic [21,22], and bladder [23] cancers by activating or suppressing different mechanisms, as shown in Figure 1.

Kaempferol is a flavonol characterized by a C6–C3–C6 backbone and the molecular formula C_15_H_10_O_6_ [24]. As a member of the flavonol subclass, it possesses a 3-hydroxyflavone skeleton [25]. The structural diversity of kaempferol relative to other flavonoids arises from differences in the position of the benzenoid substituent, the nature of the six-membered heterocyclic ring fused to the benzene ring, and the specific arrangement and bonding of carbon, hydrogen, and oxygen atoms [24]. Among flavonols, kaempferol is distinguished by the hydroxylation pattern at the 3-, 5-, 7-, and 4′-positions [11]. The biological activities of kaempferol are largely determined by the specific substitution pattern and degree of hydroxylation within its A and C rings. In particular, 5,7-dihydroxyl substitution on the A ring, together with the C2=C3 double bond and the 4-oxo group in the C ring, plays a crucial role in suppressing the expression of adhesion molecules and mediating anti-inflammatory effects. Furthermore, the hydroxyl substituents on the B and C rings are essential for the antioxidant properties of kaempferol [24].

The phytochemical structures of flavonoids are of crucial importance for their exhibited anticancer activities. For instance, glycosylated flavonoids such as rutin (quercetin-3-O-rutinoside) and prunin (naringenin-7-O-glucoside) possess sugar moieties that enhance aqueous solubility and attenuate off-target toxicity. However, these compounds frequently require deglycosylation or nanocarrier-assisted delivery systems to achieve cytotoxic intracellular concentrations [26,27]. On the other hand, methylated flavonoids such as isorhamnetin (the 3′-O-methylated derivative of quercetin) trade a portion of their polarity for enhanced cellular uptake and metabolic resistance. This characteristic has been linked to the higher cytotoxic potential observed in methoxylated flavones compared to their hydroxylated counterparts [28]. Moreover, kaempferol possesses a hydroxylation pattern that supports a wide range of versatile target interactions via direct membrane permeability.

Although the regulation of PI3K/AKT/mTOR signaling and its downstream cell death programs shows broad similarities across flavonoid groups, these effects remain qualitatively distinct. For instance, isorhamnetin suppresses PI3K/AKT/mTOR and AMPK/mTOR signaling to trigger caspase-dependent intrinsic apoptosis, elevate the BAX/BAK to BCL-2 ratio, promote cytochrome c release, and resensitize doxorubicin-resistant breast cancer through ROS-induced mitochondrial dysfunction [28]. Rutin induces apoptosis and autophagy, promotes cell cycle arrest, and is capable of modulating oxidative stress as well as tumor suppressor signaling. Structurally related flavonols such as myricetin, on the other hand, inhibit PI3K/Akt/mTOR to simultaneously induce apoptosis and protective autophagy characterized by elevated Beclin-1 and LC3-II levels [29,30]. Through this mechanism, kaempferol interacts across a remarkably broad spectrum and inhibits PI3K/AKT/mTOR in multiple tumor types. Furthermore, it activates autophagic cell death via Beclin-1 and LC3-II expression while triggering caspase-9/3-mediated intrinsic apoptosis. These compounds can also vary in their capacity to modulate epithelial–mesenchymal transition (EMT), angiogenesis, and drug resistance, contingent upon their chemical structures. Isorhamnetin reverses EMT by inhibiting AKT/ERK and AKT/GSK-3β signaling, thereby restoring E-cadherin expression while downregulating N-cadherin, vimentin, and Snail. Additionally, it attenuates angiogenesis by reducing VEGF and MMP-2/9 levels through the suppression of PI3K/AKT and STAT3 [28]. In lung cancer, kaempferol inhibits TGF-β1-driven EMT and MMP-2, suppresses VEGF/VEGFR-2-mediated angiogenesis, and reverses multidrug resistance by modulating P-glycoprotein and PKM2-dependent glycolysis. Notably, it achieves these effects through a single aglycone structure, without requiring glycosidic or methyl modifications [11].

As outlined above, the functional differences between kaempferol and other flavonoids directly stem from how a sugar moiety alters the physicochemical trajectory of the molecule. The conjugation of a hydrophilic sugar markedly enhances both aqueous solubility and chemical stability. This improves distribution within the intestinal lumen and can elevate the oral bioavailability of the parent glycoside compared to its poorly soluble aglycone counterpart [31,32]. This same hydrophilic nature opposes passive diffusion through the lipid bilayer. Flavonoid glycosides are generally too polar to cross membranes freely. Conversely, lipophilic aglycones such as kaempferol readily enter cells via passive diffusion [31]. In line with this phenomenon, aglycones exhibit superior cellular uptake and transport efficiency in intestinal epithelial models compared to their corresponding mono- or diglycosides [33]. Due to these physicochemical properties, kaempferol exhibits higher lipophilicity and cellular uptake than its glycosylated counterparts (e.g., rutin and prunin), which often rely on deglycosylation or nanocarrier formulations. This favorable membrane permeability likely facilitates sufficient intracellular bioavailability to modulate downstream cellular readouts associated with the PTEN/PI3K/AKT/mTOR pathway [26,27].

Taken together, these characteristics indicate that kaempferol possesses distinct mechanistic features that set it apart from other flavonoid-based anticancer candidates. Rather than favoring a single mode of cell death, kaempferol has the potential to concurrently activate both autophagic and apoptotic cell death through simultaneous PI3K/AKT/mTOR inhibition and Beclin-1/LC3-II induction. Furthermore, its capacity to restore expression of the tumor suppressor protein PTEN and reverse P-glycoprotein-mediated drug resistance readily distinguishes it from other compounds [28]. Furthermore, kaempferol has been reported to reverse chemoresistance across various cancer types, specifically by modulating multiple molecular pathways and disrupting metabolic and lysosomal homeostasis [34].

Non-coding microRNAs are crucial RNA molecules involved in the regulation of protein synthesis. They inhibit the translation of target mRNAs and function as negative regulators of their respective pathways. Furthermore, microRNAs have been characterized as master regulators connecting mitochondrial function, metabolic reprogramming, and chemoresistance across various malignancies [35]. Although microRNAs can act as either oncogenes or tumor suppressors, their expression levels in cancer cells can be altered by the administration of anticancer agents. Flavonoid interventions have been reported to reshape these microRNA networks, thereby triggering cell death in tumor cells [36]. Flavonoids are thought to target the PI3K/AKT/mTOR pathway, potentially through the mediation of associated microRNAs. However, in the existing literature, the effects of kaempferol on the PI3K/AKT/mTOR signaling cascade and on related microRNAs have mostly been addressed independently. In this context, this review provides a comprehensive overview of how kaempferol modulates the PTEN/PI3K/AKT/mTOR axis across various cancer types, detailing its regulatory effects on the microRNAs governing this pathway.

## 2. Herbal Sources of Kaempferol

Kaempferol is naturally synthesized in many plants, especially vegetables and fruits. The biological activities of kaempferol and its derivatives derived from these plants were assessed. Some plants that contain kaempferol and its derivatives are listed in Table 1.

## 3. The Biological Activities of Kaempferol

The unique structural architecture of kaempferol and its derivative compounds is fundamentally responsible for the biological activity it exhibits. Below, some biological activities demonstrated by kaempferol are listed.

### 3.1. Antioxidant Activity

Antioxidant activity is widely recognized as one of the prominent functions shown by phenolic compounds [4]. The antioxidant activity of kaempferol has been measured using different methods in previous studies. According to the findings of Park et al. (2006) [58], it was shown that kaempferol exhibits a significant level of in vitro DPPH (1,1-dipheniyl-2′picrylhydrazyl) radical scavenging action. In another study, kaempferol 3-O-α-L-(4-acetyl)rhamnopyranoside-7-O-α-L-rhamnopyranoside, kaempferol 3-O-α-L-(4-acetyl)rhamnopyranoside-7-O. It was determined that α-L-rhamnopyranoside and kaempferol 3-O-α-D-glucopyranoside-7-O-α-L-rhamnopyranoside showed weak activity when compared to vitamin C used as a control in the DPPH test [59]. Furthermore, in antimycin A-induced osteoblast-like MC3T3-E1 cells, kaempferol decreased mitochondrial superoxide levels [60]. Baňas et al. (2023) [61] reported that the administration of kaempferol prevents oxidative stress by suppressing mitochondrial ROS, thereby improving sperm quality. Furthermore, the DNA damage and lipid peroxidation-inhibiting activities of kaempferol-3-O-alpha-L-rhamnoside have been demonstrated using plasmid and erythrocyte models, respectively [62].

### 3.2. Anti-Inflammatory Activity

Inflammation, a protective mechanism that protects the living organism against tissue damage, is a physiological response to physical, chemical or mechanical trauma [12]. Nevertheless, the elevation of several components associated with inflammation has the potential to induce the development of illnesses, including cancer, if left uncontrolled. Previous studies have documented that mice subjected to cold stress subsequent to Kaempferol administration demonstrate anti-inflammatory properties through the inhibition of cytokines IL-9 and IL-13 and the upregulation of anti-inflammatory cytokines [63]. Moreover, Kaempferol inhibited the levels of proinflammatory cytokines TNF-, IL-1, and IL-8 and translocation of cytotoxin-associated gene A and vacuolating cytotoxin A in inflammation induced by Helicobacter pylori infection [64]. Additionally, kaempferol exhibited anti-inflammatory activity by reducing the level of inflammatory factors nitric oxide synthase (iNOS), cyclooxygenase-2 (COX-2), reactive C-protein (CRP), and nuclear factor kappa B (NF-κB) in human hepatocyte-derived cells [65]. In a different neuroinflammation model, kaempferol-3-O-β-D-glucuronate was shown to exhibit anti-neuroinflammatory activity by inhibiting the expression of NF-κB, p-JNK, p-ERK, and p-p38 MAPK, and by upregulating Nrf2/HO-1 proteins [66].

### 3.3. Antidiabetic Activity

Diabetes mellitus is a metabolic disorder that annually impacts millions of individuals across the globe. It is estimated that approximately 700 million individuals will receive a diabetes diagnosis by the year 2045 [67]. Research on natural substances has demonstrated a reduction in blood glucose levels and an improvement in insulin resistance. It was reported that kaempferol abolished the harmful effects of diabetes. It has been suggested that kaempferol protects cells against high sugar by increasing ERK phosphorylation and vasohibin-1 levels in retinal ganglion cells exposed to high sugar [68]. Furthermore, it has been observed that kaempferol has the ability to decrease blood glucose and serum HbA1c (hemoglobin A1c) concentrations, while also mitigating insulin resistance in a diabetes-induced model utilizing C57BL/6J mice that were given a high-fat diet [69]. The effects of diabetic nephropathy, diabetic retinopathy, and diabetic cardiomyopathy that occur as a result of diabetes can be inhibited by kaempferol [70]. In a streptozotocin–nicotinamide (STZ-NA)-induced diabetic mouse model, blood glucose levels were reported to significantly decrease following the oral administration of kaempferol. The same study also reported that the activity of alpha-amylase, one of the primary target proteins for mitigating elevated glucose levels, was reduced after kaempferol treatment [71].

In another study, it was reported that kaempferol accelerated the wound healing process in a designed wound study with diabetic and non-diabetic mice [2]. After the application of ointment containing kaempferol, hydroxyproline levels and reepithelialization increased in both excisional and incisional diabetic and non-diabetic wounds.

### 3.4. Antifibrotic Activity

It was reported that kaempferol administration in bleomycin-induced mice reduces skin fibrosis by suppressing oxidative stress markers such as HO-1 (Heme oxygenase 1) and NOX2 (NADPH oxidase 2) and inflammation markers such as IL-6, TNF-α (Tumor necrosis factor-α), and transforming growth factor beta (TGF-β) [72].

### 3.5. Liver Protecting Activity

Moreover, kaempferol was determined that it ameliorated acute alcoholic liver injury by increasing the expression of butyrate receptors and tight junction proteins in the intestinal mucosa [73]. In a similar study, it was determined that kaempferol might be used in the treatment of acute liver failure by decreasing C/EBP-homologous protein (CHOP) expression, as well as reducing the increase in glucose-regulated/binding immunoglobulin protein 78 (Grp78) protein and endoplasmic reticulum-stress-induced apoptosis [74].

### 3.6. Neuroprotective Activity

Hussein et al. (2018) reported that kaempferol exhibits neuroprotective activity in brain tissue by modulating the GSK3-β (Glycogen synthase kinase-3 beta) and Nrf2 (Nuclear factor erythroid 2-related factor 2) signaling pathway [75].

### 3.7. Bone Protective Activity

Adhikary et al. (2018) determined in their study that kaempferol reduces glucocorticoid-induced bone loss and increases the regeneration of damaged bone [76]. It has also been reported that the bone protection effect is through the regulation of Runx2 (RUNX Family Transcription Factor 2) and Osx (osterix) expression. It has been determined that after the administration of Kaempferol, it causes a decrease in Mastitis associated with a decrease in the expression of myeloperoxidase, interleukin-2, TNF-alpha, and Angiopoietin-like protein 2 [77]. Along with the activities given above, one of the important activities of kaempferol is its anti-cancer properties. Complementing the aforementioned data, Table 2 outlines the target pathways involved in the bioactivities of kaempferol.

## 4. An Overview of Cancer

Cancer is a genomic instability-driven disease characterized by the disruption of cellular homeostasis, inhibition of programmed cell death, and uncontrolled proliferation [82]. In a healthy organism, cell division, growth, and death are maintained under stringent genetic control [83]. However, environmental mutagens such as radiation, carcinogenic chemicals, and oncogenic viruses alongside hereditary predispositions and replication errors induce mutations within the DNA [84]. The accumulation of these mutations can transform proto-oncogenes into hyperactive oncogenes and inactivate tumor suppressor genes [85]. Furthermore, this genetic damage triggers a pathological cascade that fuels tumor nourishment via angiogenesis, enables immune evasion, and ultimately drives metastasis to distant organs through epithelial–mesenchymal transition mechanisms [86].

Cancer is a highly prevalent disease worldwide, ranking second only to cardiovascular diseases in terms of mortality. Each year, millions of individuals are diagnosed with cancer and lose their lives due to cancer-related complications [87,88]. When both sexes are evaluated collectively on a global scale, lung cancer exhibits the highest incidence, followed sequentially by colorectal, prostate, and stomach cancers. From a sex specific perspective, it is reported that in 2026, breast cancer will rank first in diagnostic frequency among women, while prostate and lung cancers will lead among men. Regarding mortality rates, lung cancer is predicted to remain the leading cause of death across both sexes [89]. Concurrently, a study encompassing 36 cancer types across 185 countries identified lung cancer as the most frequently diagnosed malignancy, followed by breast, colorectal, prostate, and stomach cancers. The same study demonstrated that lung cancer also accounts for the highest number of cancer-related deaths globally [90]. Diverse molecular signaling pathways govern the process of carcinogenesis. Among these, the PI3K/AKT/mTOR [91,92], Ras/Raf/MEK/ERK [93], Wnt/β Catenin [94], and JAK/STAT [95] pathways are the most prominently recognized.

There are different approaches for the treatment of cancer, such as surgery, radiotherapy, and chemotherapy [96]. Moreover, there is an ongoing search for new compounds to augment the therapeutic outcomes of current interventions. The intended general characteristics of these anticancer pharmaceuticals encompass the decrease in viability in cancer cells, the activation of apoptosis, the prevention of invasion and metastasis, the arrest of the cell cycle, and the modification of drug resistance in cancer. By exerting these actions, they modulate the signaling pathways associated with cancer. The PTEN/PI3K/AKT/mTOR pathway is the most well-known of these pathways [97,98,99]. The PI3K/AKT/mTOR pathway has a strong correlation with receptor tyrosine kinases. The activation of receptor tyrosine kinases leads to the autophosphorylation of cytoplasmic tyrosine residues of these receptors. Consequently, the regulatory subunit p85 of PI3K is subsequently activated. The PI3K catalytic subunit P110 protein converts phosphatidylinositol-4,5-biphosphate (PIP2) to phosphatidylinositol-3,4,5-phosphate (PIP3). The PI3K pathway is negatively regulated by phosphatase and tensin homolog (PTEN) through dephosphorylation of PIP3. AKT (protein kinase B) is stimulated through phosphorylation by phosphatidylinositol 3-dependent kinase 1, which functions as a serine/threonine kinase. mTOR is recognized as the primary downstream effector of AKT. The activation of rapamycin (mTOR) can be achieved by two mechanisms: direct phosphorylation of tuberous sclerosis-2 (TSC2) by AKT or suppression of AMP-activated protein kinase (AMPK) by AKT [97,100]. By inhibiting apoptosis and autophagy, the PI3K/AKT/mTOR pathway is recognized for its role in regulating cell viability. The targeting and suppression of the PI3K/AKT/mTOR signaling pathway by kaempferol has been shown in several cancer types.

## 5. The Therapeutic Potential of Kaempferol in Cancer Treatment Through Modulation of the PTEN/PI3K/AKT/mTOR Signaling Pathway

Phytochemicals derived from plants have been identified as potential therapeutic agents for cancer [101]. The desired characteristics of these chemicals include the ability to decrease the viability of cancer cells, trigger apoptosis, halt the cell cycle, fragment DNA, decrease the levels of inflammatory cytokines, and inhibit signaling pathways associated with cancer [102]. Previous studies have demonstrated that these effects are exerted by kaempferol in various cancer cell types. Following kaempferol administration, caspase 9 and caspase 3 levels significantly increased in MDA MB 231 breast cancer cells, thereby triggering intrinsic apoptosis and inducing cell cycle arrest in the G2/M phase [15]. According to the findings of Zhang et al. (2022) [103], kaempferol exhibited anticancer properties in the androgen-dependent and androgen-independent prostate cancer cell line PC3 through the downregulation of Ki67 expression. Furthermore, kaempferol has been reported to modulate multiple molecular pathways in various malignancies [104].

A unifying thread running through kaempferol’s pleiotropic bioactivities, its antioxidant, anti-inflammatory, antifibrotic, and neuroprotective actions, is the regulation of redox homeostasis, and this places oxidative stress at the center of its potential anticancer relevance. Reactive oxygen species (ROS) are not merely byproducts of tumor metabolism but act as central regulators of the tumor immune microenvironment (TIME): ROS generated by both malignant and infiltrating immune cells reshape immune cell function, drive metabolic competition for nutrients between tumor and immune populations, and dictate the balance among regulated cell-death pathways, thereby promoting tumor progression and immune evasion [105]. Because kaempferol modulates cellular ROS as part of its core pharmacology, it is mechanistically positioned to influence this oxidative stress axis of the TIME. Depending on dose and context, a flavonoid can lower ROS to protect anti-tumor lymphocytes from oxidative exhaustion or, conversely, raise ROS in malignant cells to favor immunogenic cell death. Therefore, the redox activity of kaempferol should be considered not only for its direct effects on tumor cell signaling but also for its ability to remodel the immune microenvironment that supports the development of PI3K/AKT/mTOR-driven tumors [105].

The anticancer activity of kaempferol has been attributed to its modulation of the PI3K/AKT/mTOR signaling pathway in various types of cancer [61]. Kashafi et al. (2017) [106] demonstrated that the treatment of kaempferol suppressed the PI3K/AKT and hTERT signaling pathways, thereby inducing apoptosis and cellular senescence. Furthermore, kaempferol has been shown to inhibit angiogenesis, a process that supports tumor growth and progression, through the suppression of the PI3K/AKT, MEK, and ERK signaling pathways, as well as the regulation of vascular endothelial growth factor receptor-2 (VEGFR-2) [107].

The mechanistic effects of kaempferol on the PI3K/AKT/mTOR signaling pathway remain incompletely understood. Nevertheless, accumulating evidence indicates that kaempferol upregulates phosphatase and tensin homolog (PTEN) expression. PTEN is a critical tumor suppressor that plays a pivotal role in inhibiting cell growth and enhancing cellular susceptibility to apoptosis (programmed cell death). It also serves as a key negative regulator of the PI3K/AKT/mTOR signaling axis. Kaempferol has been shown to increase PTEN expression in a time-dependent manner, leading to a reduction in AKT phosphorylation at Ser473 [108]. Through its lipid phosphatase activity, PTEN decreases AKT phosphorylation, thereby relieving the inhibitory effects of AKT on the pro-apoptotic proteins BAD and FOXO, ultimately promoting apoptosis. In contrast, phosphorylation and activation of the PI3K p110 catalytic subunit stimulate downstream AKT and mTOR signaling. Molecular docking analyses have further demonstrated that flavonoids can bind to PI3K by interacting with catalytically important amino acid residues, including Lys833 and Asp964, suggesting a potential mechanism by which kaempferol may suppress PI3K activity [109].

**Figure 2:**
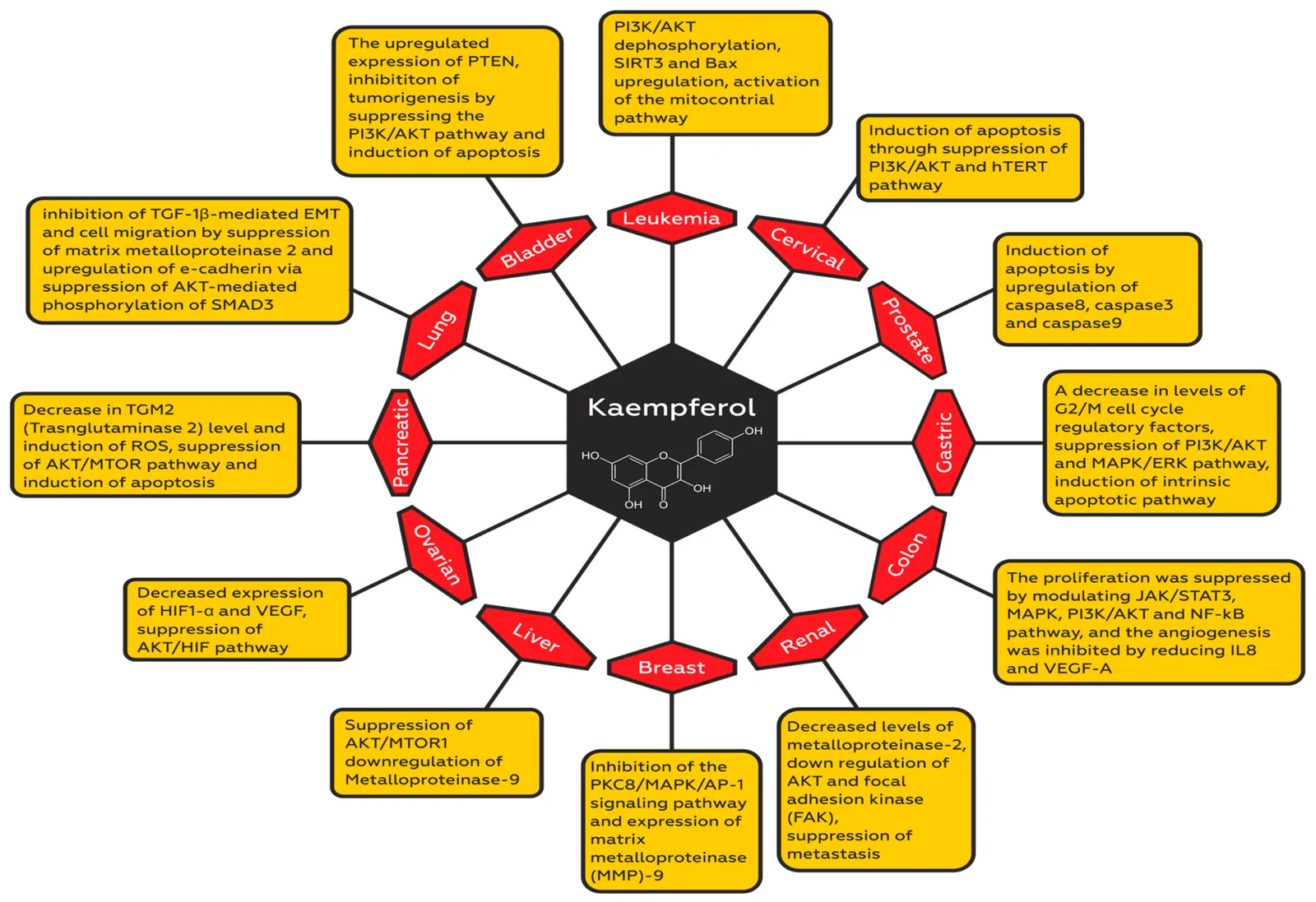
Effects of kaempferol on PI3K/AKT/mTOR pathway in different cancer cells.

The following sections provide an overview of the effects of kaempferol treatment on the PI3K/AKT/mTOR signaling pathway in various cancer cell types. The effects of kaempferol on the PI3K/AKT/mTOR pathway in different cancer cells are ordered and summarized in Figure 2.

**Pancreatic Cancer:** Pancreatic cancer is among the most aggressive forms of the disease. It has been demonstrated that inhibiting the PI3K/AKT/mTOR pathway is crucial for preventing pancreatic cancer progression [110]. According to the findings of Wang et al. (2021) [22], the administration of kaempferol to pancreatic cells (PANC1 and MIA PaCa-2 cells) led to a decrease in KEAP1 (Kelch-like ECH-associated protein 1) level, which is an adaptor subunit of Cullin 3-based E3 ubiquitin ligase) and an increase in Nrf2 (nuclear factor-erythroid 2 p45-related factor 2) level. As a result, subsequent to kaempferol application, TGM2 (Transglutaminase 2), a protein that normally exhibits upregulation and inhibits reactive oxygen species (ROS), decreases in both cell types. This decrease in TGM2 leads to an increase in ROS levels, which subsequently triggers apoptosis and inhibits the AKT/MTOR pathway [22]. In a different study, the combination of kaempferol and sulfatinib was reported to inhibit pancreatic neuroendocrine tumor cells mediated by the suppression of the PI3K/AKT/mTOR pathway. This study further demonstrated that cell proliferation was inhibited through the suppression of Ki-67, angiogenesis was prevented via the suppression of VEGFA and CD31, and apoptosis was induced [111]. Sugita et al. (2026) [112] reported that in gentamicin-resistant pancreatic cancer cells, kaempferol administration inhibited cell proliferation and migration by blocking the Akt and NF-κB signaling pathways and decreasing MMP-1 protein levels.

**Bladder Cancer:** Xie et al. (2013) [113] reported that kaempferol stimulates the expression of PTEN, a tumor suppressor protein, and inhibits tumorigenesis of human bladder cancer cells by suppressing the PI3K/AKT pathway and inducing apoptosis.

**Gastric Cancer:** Song et al. (2015) [19] demonstrated that proliferation of gastric cancer cells was significantly inhibited in relation to the suppression of AKT, ERK, and COX (cyclooxygenase) signaling pathways after the application of kaempferol. Furthermore, it has been indicated that an upregulation of proapoptotic proteins Bax, caspase-3, and caspase9, accompanied by a downregulation of anti-apoptotic protein Bcl-2, induces apoptosis. Additionally, a reduction in the levels of G2/M cell cycle regulatory factors, including cyclin B1, Cdk1, and Cdc25C, induces cell cycle arrest. Kaempferol treatment of gastric cancer cells inhibited the PI3K/AKT and MAPK/ERK signaling pathways and prevented cell proliferation. Gao et al. (2025) [114] reported that the administration of kaempferol to gastric cancer cells inactivated the AKT/GSK3β signaling pathway and suppressed the expression of invasion-related genes (SRC, MMP9, CXCR4, KDR, and MMP2), thereby inhibiting the proliferation, migration, and invasion of cancer cells.

**Leukemia:** Marfe et al. (2009) [115] reported that the apoptosis activated by an increase in expression of SIRT3 and Bax, release of cytochrome C, and activation of Caspase 3 in relation to the inactivation of PI3K and dephosphorylation of AKT in chronic myelogenous leukemia cell line K562 and promyelocytic human leukemia U937 cells after the application of kaempferol.

**Lung Cancer:** Kaempferol has been demonstrated to inhibit cell migration and TGF-1β-mediated epithelial–mesenchymal transition (EMT) in lung cancer by inhibiting matrix metalloproteinase 2, and to upregulate e-cadherin by inhibiting AKT-mediated phosphorylation of SMAD3 [116]. Nguyen et al. [20] reported that mitogen-activated protein kinase (MAPK) was activated in lung cancer cells treated with kaempferol, whereas Akt-1 and phosphorylated Akt-1 were inhibited, and the activation of poly ADP-ribose polymerase (PARP) and caspase-7 was found to induce apoptosis. On the other hand, phosphorylation of p38 and JNK kinases, which are involved in apoptosis induction, was demonstrated to decrease following kaempferol treatment; apoptosis was induced exclusively via activation of the MEK/MAPK pathway. In their study, Wang et al. (2023) [117] observed that kaempferol treatment resulted in a reduction in cell viability, inhibition of the PI3K/AKT/mTOR pathway, and activation of the autophagy death pathway in A549 and H1299 cells. The same study demonstrated that the autophagy markers Beclin 1 and LC3-II increased, while p62 levels decreased following kaempferol administration. In another study, kaempferol treatment in primary lung adenocarcinoma cells generated by conditional reprogramming technology was shown to upregulate E-cadherin expression by inhibiting the IGF-1R/PI3K/AKT signaling pathway, and to partially reverse the epithelial–mesenchymal transition (EMT) by reducing vimentin and alpha-SMA levels, thereby suppressing tumor cell proliferation and invasion [118].

**Renal Cancer:** Hung et al. (2017) [119] determined that the application of kaempferol to renal cancer cells (786-O renal cancer cells) resulted in the suppression of MMP2 expression and the inhibition of invasion and migration of these cells via the phosphorylation of focal adhesion kinase (FAK) and AKT. In the same study, however, no alterations were detected in the ERK and JNK/p38 pathways.

**Cervical Cancer:** Previous studies have demonstrated that kaempferol inhibits the proliferation of cervical cancer cells and enhances their susceptibility to multidrug resistance. According to a study by Limtrakul et al. (2005) [120], kaempferol inhibited the proliferation of the multidrug-resistant human cervical cancer cell line KB-V1, which has a high expression of P-glycoprotein, by modulating MDR. Furthermore, a reduction in the expression level and functionality of P-glycoprotein was identified, leading to a corresponding decrease in the efflux of vinblastine, a substance used to treat cancer, from cancer cells. An alternative report suggested that kaempferol administration induced apoptosis in cervical cancer cells via inhibition of the PI3K/AKT and hTERT (Human telomerase reverse transcriptase) pathways [106]. Choi et al. (2024) [121] demonstrated that following kaempferol administration in cervical cancer cells, cell viability and migration were suppressed through the inhibition of the PI3K/AKT/mTOR pathway, while apoptotic and autophagy-related proteins were upregulated.

**Prostate Cancer:** Prostate cancer is one of the most prevalent malignancies affecting males. Previous studies have documented that kaempferol inhibits PCNA (Proliferating cell nuclear antigen) and VCAM1 (Vascular Cell Adhesion Molecule 1), thereby decreasing the viability of prostate cancer cells [122]. Furthermore, Wang et al. (2021) [123] documented that the treatment of kaempferol effectively inhibited the progression of benign prostatic hyperplasia in rats. A different investigation demonstrated that the application of kaempferol-3-O-rhamnoside to LNCaP human prostate cancer cells inhibited proliferation and induced apoptosis via the upregulation of apoptotic caspase-8, caspase-3, and caspase-9. Nevertheless, to the best of our knowledge, the literature lacks sufficient information about the PTEN/PI3K/AKT/mTOR pathway in prostate cancer studies after kaempferol administration.

**Colon Cancer:** The regulation of acquired drug resistance is crucial for effective cancer treatment. When administered alone or in combination with anticancer drugs, kaempferol was applied to drug-resistant LS174 colon cancer cells, the proliferation was suppressed by modulating the JAK/STAT3, MAPK, PI3K/AKT, and NF-kB pathways, and angiogenesis was inhibited by reducing IL8 and VEGF-A [124]. Li et al. (2019) [125] reported that the combined administration of kaempferol and 5-fluorouracil increased Bax levels, decreased Bcl-2 levels, and induced apoptosis in colorectal cancer cells (HCT 8 or HCT 116). CRC cell proliferation might have been synergistically inhibited by kaempferol and 5-FU via inhibition of the PI3K/AKT pathway. In a different study, kaempferol was demonstrated to comprehensively suppress the proliferation, migration, and invasion of hypoxic colon cancer cells by simultaneously modulating HIF-1α-, VEGF-, ANG1-, and VEGFR2-mediated angiogenesis; the Wnt pathway operating through beta-catenin, c-Myc, Cyclin-D1, LEF1, APC, and Axin-2 proteins; EMT progression via the regulation of E-cadherin, N-cadherin, Vimentin, and MMP-2/9; and cleaved caspase-3/9 and Bcl-2-dependent intrinsic apoptosis through p-ERK/p-38 MAPK signaling [126]. Selvakumar et al. (2026) [127] demonstrated through network pharmacology and molecular dynamics simulation analyses that apigenin and kaempferol target colon cancer-associated hub genes, exhibiting a structurally stable inhibitory potential by binding particularly to the AKT1 protein, which has a high mutation frequency with strong binding energies of −9.4 and −9.2 kcal/mol, respectively.

**Ovarian Cancer:** Previous studies have documented that kaempferol inhibits the growth of ovarian cancer cells. After treatment with kaempferol, Luo et al. (2009) [128] demonstrated that VEGF (vascular endothelial growth factor) expression and cell viability of OVCAR-3 and A2780/CP70 ovarian cancer cells decreased. The expression level of HIF1-α (Hypoxia-inducible factor alpha), a VEGF regulator, was similarly inhibited following treatment with kaempferol in the same study. It was also reported to inhibit the AKT/HIF pathway and suppress AKT phosphorylation at concentrations ranging from 5 to 20 μM.

**Breast Cancer:** Breast cancer is the most common cause of death among women after lung cancer. It has been shown that proliferation of breast cancer cells is suppressed, apoptosis is induced, and the cell cycle is arrested after kaempferol treatment [15]. Previous research conducted by Wang et al. (2019) [14] has demonstrated the inhibitory effect of kaempferol on the growth of MDA-MB-453 cells. This effect was seen through the suppression of CDK1, resulting in the arrest of breast cancer cells in the G2/M phase. It has been reported that kaempferol inhibits the invasion of breast cancer cells by obstructing the PKC/MAPK/AP-1 cascade, which is responsible for the subsequent expression and activity of matrix metalloproteinase (MMP)-9. After treatment with kaempferol, the levels of the EMT markers vimentin, snail, and slug were observed to increase, while the levels of MMP2/MMP9 decreased in triple-negative breast cancer cells. PI3K/AKT/mTOR signaling pathway dysregulation is a frequent occurrence in breast cancer cells. This promotes the proliferation of breast cancer cells by causing mutations in tumor suppressor genes, including INPP4B and PTEN phosphatases, and increased PI3K activity or loss of PI3K inhibitory functions.

In a study utilizing breast cancer cells, Jamshidi et al. (2025) [129] demonstrated through molecular docking and molecular dynamics simulations that the compounds kaempferol 3-rutinoside-4′-glucoside and kaempferol 3-rutinoside-7-sophoroside suppress MCF-7 cell proliferation and trigger apoptosis by upregulating FOXO3 expression, binding to the ATP-binding site of Akt1 with extraordinary affinities of −21.79 and −20.73 kcal/mol, respectively.

**Liver Cancer:** Liver cancer is considered one of the deadliest malignancies, accounting for a significant number of fatalities annually, resulting in a significant number of fatalities annually. The work conducted by Ju et al. (2021) [130] demonstrated the suppression of matrix Metalloproteinase-9 and inhibition of AKT phosphorylation following kaempferol administration in human HCC cancer cells. According to findings from another study, SK-HEP-1 cells treated with kaempferol showed increased levels of autophagic markers such as p-AMPK, LC3-II, Atg 5, Atg 7, Atg 12, and beclin 1, while CDK1, cyclin B, p-AKT, and p-mTOR protein levels were inhibited, and autophagic cell death was triggered [131]. In an in silico investigation, Siniprasad et al. (2020) [132] showed that kaempferol markedly inhibited both mTORC1 and AKT. Furthermore, kaempferol treatment inhibited migration and invasion by blocking the PI3K/AKT/MMP2/MMP9 pathway and reduced proliferation in liver cancer cells by triggering the mitochondrial signaling pathway [70]. In a study conducted by Han et al. (2017) [133], it was demonstrated that the administration of kaempferol resulted in the activation of AMP-activated protein kinase (AMPK) in human hepatocellular carcinoma (HCC) cells. AMPK is recognized as the principal energy-sensing mechanism in mammalian cells. The study showed that kaempferol has the ability to elevate the expression of p53 protein and inhibit the activity of the rapamycin (mTOR) complex 1 (mTORC1), while also stimulating the autophagy pathway through the activation of AMPK in hepatocellular carcinoma (HCC) cells.

## 6. Structure–Activity Relationship (SAR) of Kaempferol

**Figure 3:**
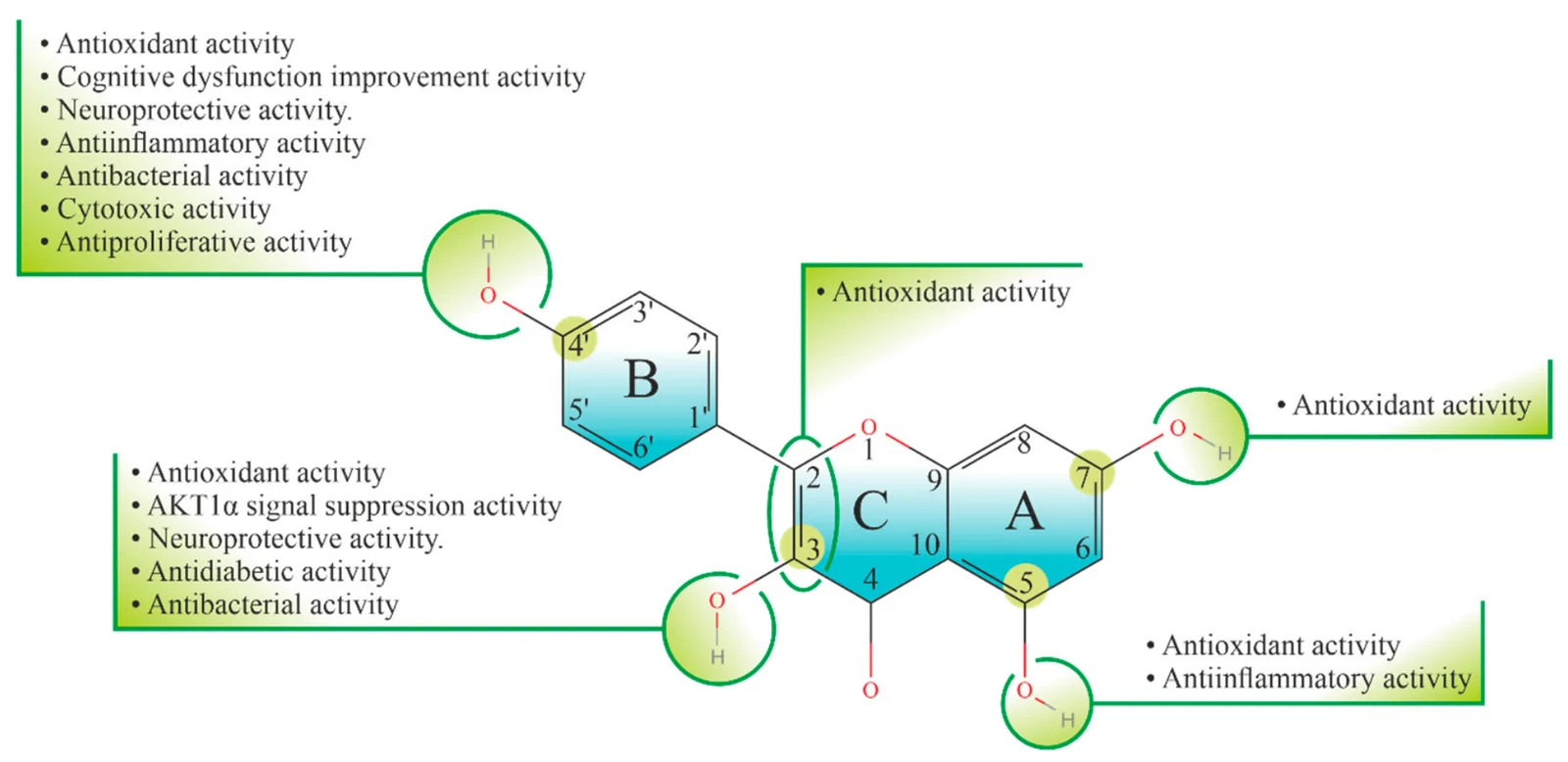
The chemical structure of kaempferol and the activities associated with this structure.

Structural modifications across the A, B, and C rings impart unique physicochemical properties to kaempferol, enabling it to exhibit versatile biological activities. The chemical structure of kaempferol and the activities associated with this structure are summarized in Figure 3. The number and position of the OH groups in kaempferol play a significant role in its bioactivity.

The most well-known of these activities is antioxidant activity. Kaempferol has a diphenylpropane (C6-C3-C6) backbone containing four hydroxyl substituents, which enables it to scavenge free radicals in association with significant redox activity [134]. Sarian et al. (2017) [135] showed that OH groups play important roles in exhibiting antioxidant activity, but this depends not only on the excess of OH groups but also on the configuration of hydroxylation. For example, kaempferol, which contains only 4-OH groups (3, 5, 7, and 4′), has been reported to show higher DPPH and FRAP activity when compared to flavanols and flavones containing 5-OH groups, such as (+)-catechin (3, 5, 7, 3′, and 4′), (−)-epicatechin (3, 5, 7, 3′, and 4′), and hypolaetin (5, 7, 8, 3′, and 4′). However, different chemical bonds of kaempferol can cause changes in antioxidant activity. For example, Chear et al. (2019) [136] reported that di-acylation at the 3″-O and 6″-O positions of kaempferol 3-O-β-D-glucopyranoside increased both its free radical scavenging and cytotoxic properties, while mono-acylation at both positions did not alter the activity. The study showed that feruloyl acylation of kaempferol 3-O-(3″-O-E-p-coumaroyl)-(6″-O-E-feruloyl)-β-D-glucopyranoside and kaempferol 3-O-(3″, 6″ di-O-E-p-coumaroyl)-β-D-glucopyranoside exhibits more effective free radical scavenging activity compared to coumaryl acylation. Similarly, while kaempferol aglycone demonstrated potent radical scavenging capacity in the DPPH assay, its glycosylated derivatives such as kaempferol 3-O-β-d–glucopyranosyl (1→2) β–d-xylopyranoside and kaempferol 3-O-α-l-arabinopyranosyl (1→2) β-d-galactopyranoside) exhibited significantly lower antioxidant activities [137]. Nevertheless, the presence of the C2=C3 double bond in the C-ring remains a key structural feature driving enhanced antioxidant activity.

In addition to its antioxidant capacity, kaempferol exhibits significant anti-inflammatory properties. Kaempferol inhibits the expression levels of inflammation-related cytokines and enzymes, such as TNF-α, NO, iNOS, and COX. It has been suggested that the 4′-OH group in the B ring and the 5′-OH group in the A ring of kaempferol enhance the anti-inflammatory activity, whereas the 3′-OH group on the C ring may atteunate this effect. Furthermore, the absence of the 6′-OH group on the A ring and the 3′-OH group on the B ring play a crucial role in exerting this activity [138]. Wei et al. (2026) [139] showed that kaempferol increased the level of proapoptotic protein BAX level and decreased the level of BCL2 in a rheumatoid arthritis model. In the same study, molecular docking analyses revealed that kaempferol interacts with AKT1, PIK3R1, and HSP90AA1 proteins, with binding energies reported as −6.51, −4.26, and −6.51 kcal/mol, respectively. In a different study, the PI3Kα inhibitory activity of kaempferol, quercetin, myricetin, quercetagetin, and quercetin 3-β-D-glucoside was demonstrated. Among the tested flavonoids, the weakest effect was exhibited by kaempferol after quercetin 3-β-D-glucoside, but the activity increased with increasing concentration. This activity was reported to positively correlate with the number of hydroxyl groups on both the A and B rings. On the other hand, the number of hydroxyl groups on the C ring was found to have no PI3Kα inhibitory activity [140].

In association with its antioxidant and anti-inflammatory properties, kaempferol has been demonstrated in vivo to improve cognitive impairment and exhibit neuroprotective effects. In mice, kaempferol administration is considered to alleviate cognitive dysfunction by activating the cAMP/ERK1/CREB signaling pathway, increasing Na^+^/K^+^-ATPase enzyme activity, and reducing hippocampal oxidative stress. Furthermore, in D-galactose-induced mouse models, an increase in the number of hydroxyl groups on the flavonol B ring has been shown to attenuate cognitive decline by increasing ERK1/CREB protein expression [141]. In another study, Yan et al. (2019) [142] demonstrated that in mice treated with Kaempferide, formed by methylation of the 4′-OH in the B ring of kaempferol, neurons were arranged in an integrated and orderly manner, and the compound could induce brain-derived neurotrophic factor (BDNF)/tropomyosin receptor kinase B (TrkB)/cAMP response element binding (CREB) protein signaling in the hippocampus. It was reported that Kaempferide exerts neuroprotective effects in an Aβ1–42-induced mouse model by mitigating oxidative stress and enhancing the BDNF/TrkB/CREB pathway. Hydroxyl and methoxy groups in flavonoids can play a pivotal role in anticholinesterase activity, which can be enhanced by the presence of hydroxylated forms at the C3′ and C5′ positions of the core skeleton. Kaempferol exhibits inhibitory activity against acetylcholinesterase by forming two hydrogen bonds with the Phe295 and Ser293 residues of the enzyme. Conversely, glycosylation at positions 3 and 7 of the C ring in kaempferol has been shown to exert a weak effect on anticholinesterase activity [143].

The addition of a hydroxyl (–OH) group at the C3 position of the C ring in kaempferol exerts antidiabetic effects by inhibiting α-glucosidase and α-amylase activities [143]. The effect of kaempferol on α-glucosidase, an enzyme located in the epithelium of the small intestine that catalyzes the breakdown of disaccharides into monosaccharides, has been elucidated. It is suggested that the dihydroxyl groups at the C-3 and C-4 (catechol) positions of flavonoids effectively conjugate with the active site residues of α-glucosidase, a process further facilitated by the presence of the catechol moiety on the B ring. Conversely, because kaempferol possesses only a single hydroxyl group on its B ring and lacks a catechol system, it has been reported to exhibit reduced interaction with the binding site residues of α-glucosidase. Furthermore, glycosylation at positions 3 and 7 of the C ring in kaempferol was reported to result in weak inhibitory effects against both α-glucosidase and α-amylase [135].

DNA gyrase is an essential enzyme required for bacterial replication. In flavonoids, the 3′-OH groups on the C ring and the 4′-OH groups on the B ring in flavonoids enhance antibacterial activity, whereas the 5′-OH groups on the C ring and the 6′-OH groups on the A ring decrease activity. Kaempferol exhibits antibacterial activity by inhibiting DNA gyrase, primarily mediated through the 3′-OH group of its C ring [144].

Anticancer activity represents one of the most extensively studied bioactivities of phytochemicals. Kaempferol exhibits a significant role in mediating anticancer mechanisms, including anti-proliferation, suppression of metastasis, induction of apoptosis, and cell cycle arrest. Previous studies reported that kaempferol inhibited the proliferation of HL-60 leukemia cells, arrested the cell cycle, increased caspase 3 activity, and reduced BCL2 levels [145]. Apoptosis is a programmed cell death pathway exploited to suppress cancer cell survival. One of the key enzymes involved in the modulation of apoptotic signaling is glyoxalase I. Molecular docking studies have demonstrated a direct positive correlation between the number of OH groups on the B ring of flavanols and their binding affinity to the active site of this enzyme. However, kaempferol has only one OH group in its B ring, and this binding is expected to be lower compared to other flavanols [145]. In another study, kaempferol was reported to induce apoptosis in DLD1 colorectal cancer cells by binding to the anti-apoptotic protein BCL2 via hydrogen bonds and hydrophobic interactions with the Asn140 and Tyr199 residues [146]. In some cases, specific chemical structural features enhance cytotoxic activity without altering other bioactive properties. As mentioned above, Chear et al. (2019) [136] reported that feruloyl acylation of kaempferol 3-O-(3″-O-E-p-coumaroyl)-(6″-O-E-feruloyl)-β-D-glucopyranoside and kaempferol 3-O-(3″, 6″-di-O-E-p-coumaroyl)-β-D-glucopyranoside exhibited stronger antioxidant activity than coumaryl acylation. However, in the cytotoxic activity assay conducted within the same study, coumaryl acylation was shown to exert higher cytotoxicity when compared to feruloyl acylation.

## 7. MicroRNAs as Non-Coding RNAs

MiRNAs, which negatively affect gene expression by inhibiting translation, are non-coding and small molecules (approximately 18–22 nucleotides) [70]. More than 50% of mammalian miRNA genes are located in the intronic regions of protein-coding or non-protein-coding genes. miRNAs in intergenic regions have transcriptional regulatory elements that form independent transcription units [147]. RNA polymerase II typically transcribes a pre-miRNA precursor. After that, endonuclease enzymes like DGCR8 and DROSHA cleave it in the nucleus, producing a pre-miRNA sequence made up of around 100 nucleotides. Pre-miRNAs are transported from the nucleus to the cytoplasm by the nuclear export protein Exportin-5. After the pre-miRNA is cleaved by the cytoplasmic ribonuclease Dicer, a mature double-stranded miRNA is produced. The RNA-induced silencing complex (RISC), which is formed when the miRNA attaches to Argonaute (Ago) proteins, subsequently controls the translation of complementary messenger RNA (mRNA) [148]. MicroRNAs (miRNAs) regulate of gene expression by binding partly to the non-coding region of their target mRNA, namely on the 3′ untranslated region (3′UTR). On the other hand, recent studies have shown that miRNAs can interact not only with 3′-UTR but also with 5′-UTR of target mRNAs [149]. This binding is based on complementary base pairing and typically results in the suppression of mRNA translation [150]. It has been determined that a single miRNA targets around 200 mRNAs. Recent research has demonstrated that microRNAs (miRNAs) are involved in the development of numerous diseases, including cancer [151]. This type of non-coding RNA plays a direct or indirect role in the upregulation of proteins involved in cancer-inducing pathways and suppression of tumor suppressor proteins, especially in cancer cells [152,153]. miRNAs are involved in cancer cell development, cell cycle regulation, differentiation, and apoptosis [154]. The chemicals used as antineoplastic and anticarcinogen agents have been reported to exert their activity by modulating the expression of miRNAs and their target proteins within cancer signaling pathways.

## 8. Effects of Kaempferol on Expression of miRNAs Targeting Proteins Associated with the PI3K/AKT/mTOR Signaling Pathway in Cancer Cells

Several disease models have demonstrated that kaempferol modulates the expression of miRNAs involved in diverse signaling pathways. In an atherosclerosis model, kaempferol treatment was shown to alleviate endothelial cell injury through the modulation of miR-6873-3p [155]. In another study, kaempferol activated the BMP4/BMP signaling pathway, resulting in the upregulation of miR-21 expression. Increased miR-21 expression suppressed the proliferation and migration of vascular smooth muscle cells (VSMCs) by targeting DOCK4, DOCK5, and DOCK7 mRNAs, thereby exerting cardioprotective effects [156]. Zhou et al. (2022) [157] reported that kaempferol attenuated hepatic stellate cell activation and liver fibrosis by upregulating miR-26b-5p, which in turn inhibited the Jag1/Notch signaling pathway. Furthermore, kaempferol has been shown to suppress miR-155 expression, thereby regulating the SOCS1/NF-κB signaling pathway, enhancing intestinal barrier integrity, and mitigating alcohol-induced liver injury [158].

In addition to their roles in the aforementioned pathways, miRNAs also play an important role as tumor suppressor factors. The inhibition of cancer cells can be achieved particularly through the negative regulation of signaling elements and factors involved in processes such as growth, division, migration, and mutation by suppressor miRNAs. Furthermore, various studies have reported that Kaempferol inhibits the invasion, migration, and proliferation of cancer cells by acting on different miRNAs, and eliminates cancer cells by activating apoptosis and other cell death pathways. Pu et al. (2024) [159] demonstrated that kaempferol binds to HNRNPK/HNRNPL proteins, thereby reducing the formation of circ_0000345, which is associated with an increase in miR-205-5p activity. Through this mechanism, it was shown to suppress colorectal cancer metastasis via the circ_0000345/miR-205-5p/JMJD2C/β-catenin axis. Furthermore, kaempferol was reported to increase miR-339-5p levels in colon cancer cell lines (HCT116 and DLD1); the decrease in hnRNPA1 and PTBP1, which are targets of miR-339-5p, subsequently down-regulated PKM2 (pyruvate kinase M2) levels [160]. Consequently, glycolysis, which plays a critical role in the nutrition of cancer cells, was suppressed, and colon cancer growth was inhibited [160]. In another an study, Bulut and Süslüer (2026) [161] reported that kaempferol treatment in chronic myeloid leukemia cells downregulated the expression of miR-21, miR-151-5p, miR-26b, and miR-15b, while upregulating miR-125a-5p, miR-374b, miR-19a, miR-19b, miR-222, miR-128, miR-181a, miR-210, miR-150, miR-376c, miR-196b, miR-124, miR-143, miR-28-3p, miR-122, miR-29b, miR-155, and miR-141. These alterations were accompanied by modulation of apoptosis- and cell cycle-related proteins.

Furthermore, studies on the miRNAs involved in the suppression of the PI3K/AKT/mTOR pathway by kaempferol are also available, though their number remains limited. For example, Ji et al. (2025) [162] reported that kaempferol treatment inhibited the proliferation, migration, and invasion of SK-ES-1 cells, an aggressive pediatric bone cancer model of Ewing sarcoma. In this study, kaempferol increased the expression of miR-26b-5p, which in turn reduced the expression of FAM98A (Family with sequence similarity 98 member A), a calcium regulatory protein involved in the progression of many cancer types. This regulation was associated with inhibition of the EGFR/PI3K/AKT/NF-κB signaling pathway, thereby suppressing tumor growth and metastasis. In another study, treatment of colorectal cancer cells with kaempferol-3-O-glucoside was reported to reduce the levels of oncogenic miR-31 and miR-92a, while increasing AMP-activated protein kinase (AMPK) expression [141]. Considering that active AMPK is known to inactivate mTORC1 [163], and so, it is hypothesized that the downregulation of miR-31 and miR-92a following kaempferol treatment may be associated with the PI3K/AKT/mTOR signaling axis in cancer cells. As mentioned at the beginning of this section, kaempferol can modulate miRNAs in various disease models. Moreover, it is recommended that miRNA profiling be performed following kaempferol treatment in different cancer cell lines, and that the effects of miRNAs exhibiting altered expression levels on the PI3K/AKT/mTOR signaling pathway be validated through loss-of-function and gain-of-function experiments.

**Figure 4:**
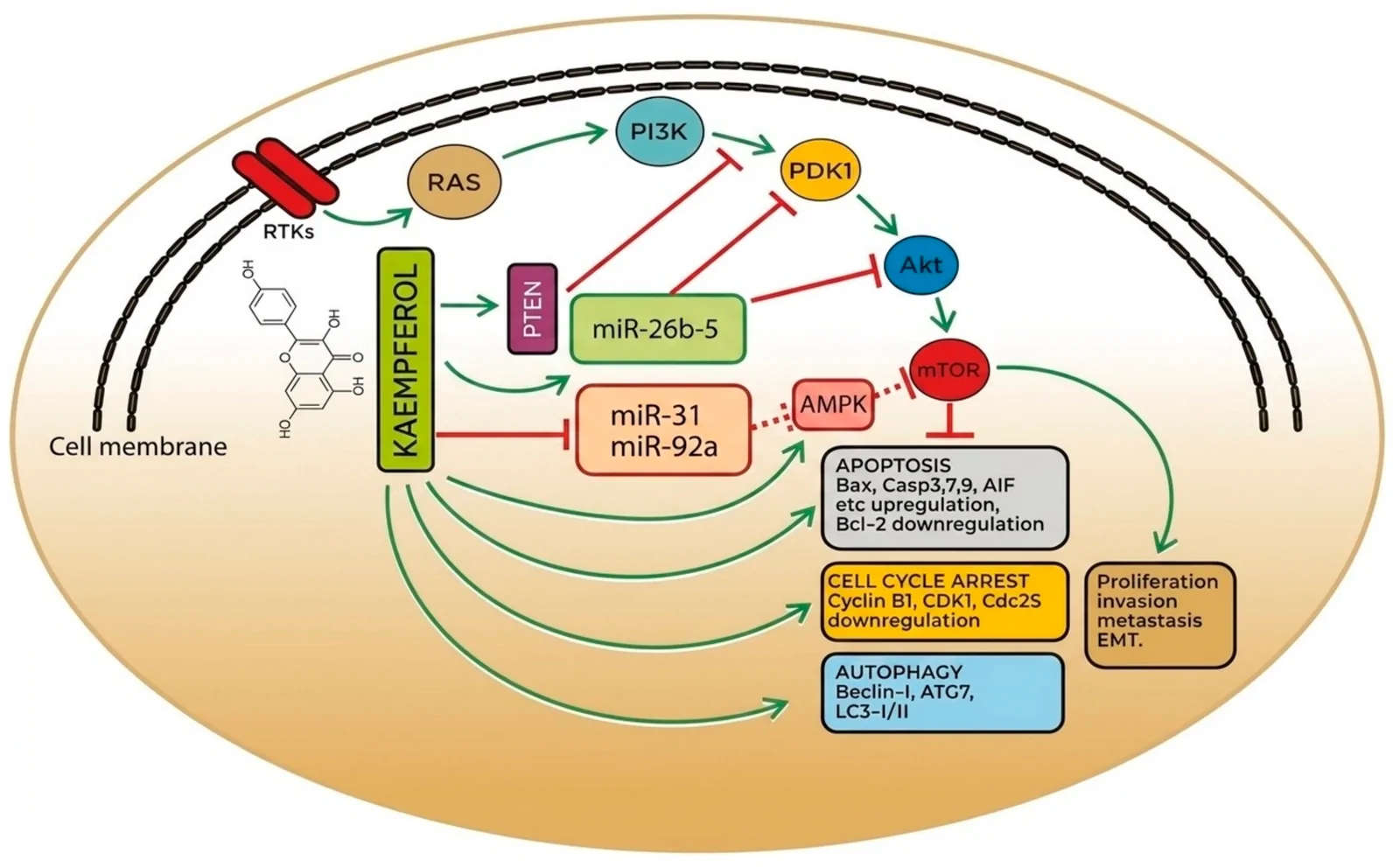
Interactions of kaempferol with miRNAs involved in the suppression and induction of the PI3K/AKT/mTOR pathway, proliferation, invasion, metastasis, and cell death pathways in different cancer cells. (Red arrows represent downregulation and green arrows represent upregulation. The dashed red arrow represents the hypothetical description of the suppression of the mechanism).

The interactions of kaempferol with miRNAs involved in the suppression and induction of the PI3K/AKT/mTOR pathway, proliferation, invasion, metastasis, and cell death pathways in different cancer cells are shown in Figure 4.

## 9. Kaempferol and Long Non-Coding RNAs (lncRNAs)

The preceding sections summarized microRNA circuits for which direct, kaempferol-specific functional evidence exists. Although studies have demonstrated the regulatory effects of kaempferol on non-coding RNAs, such as long non-coding RNAs (lncRNAs), in various experimental models [163], no studies have been reported investigating its effects on these molecules in cancer cells. Based on our literature review, no studies have demonstrated that kaempferol regulates long non-coding RNAs (lncRNAs) in cancer. Therefore, the proposals presented below should be regarded as testable hypotheses rather than established mechanisms of kaempferol action.

Long non-coding RNAs (lncRNAs), which are transcripts exceeding 200 nucleotides in length, possess important regulatory functions in fundamental biological processes, including cell growth, proliferation, differentiation, and programmed cell death, despite their inability to encode proteins. Aberrant expression profiles of these molecules play critical roles in the pathogenesis of various diseases, particularly cancer [164]. In different cancer types, lncRNAs have been shown to act as key regulators of tumor angiogenesis and epithelial–mesenchymal transition (EMT) by modulating oncogenic signaling pathways, angiogenic factors, and epigenetic mechanisms. Several lncRNAs, including MALAT1, UBE2CP3, HULC, UCA1, and PCA3, can enhance the metastatic potential of cancer cells through the regulation of gene expression [165]. Similar to miRNAs, lncRNAs can also be modulated by phytochemicals [166]. For instance, BDLNR, a lncRNA identified as a contributor to cervical cancer progression, was shown to be downregulated by baicalein. The suppression of BDLNR and its downstream target PI3K/AKT pathway by baicalein contributed to the inhibition of cell proliferation, migration, and tumor growth, as well as the induction of cell death in cervical cancer cells [167]. On the other hand, although studies investigating the effects of kaempferol on lncRNA functions in cancer cells are currently lacking, a study in osteoarthritis demonstrated the regulatory effects of kaempferol on lncRNA expression. Kaempferol treatment increased lnc-XIST expression, which subsequently modulated miR-130a expression and suppressed pro-inflammatory cytokine production and extracellular matrix degradation in C28/I2 cells [163]. Therefore, elucidating the effects of kaempferol on the expression levels and functional roles of lncRNAs, such as MALAT1, UBE2CP3, HULC, UCA1, PCA3, and XIST, in cancer cells may provide valuable insights into the identification of novel molecular targets and the development of potential therapeutic strategies for cancer treatment.

## 10. Conclusions and Recommendations

For many years, many herbal materials and compounds have been used in studies on the suppression of cancer cells. However, today, flavonoid-derived compounds with known pharmacological effects are considered promising for therapeutic investigation. In this review, the effects of kaempferol, a flavonoid-derived compound, on the PI3K/AKT/mTOR pathway and related proteins that stimulate proliferation, migration, and invasion of various cancer cells were discussed. Thanks to the chemical structure of kaempferol, it is thought that proteins in this pathway can either be directly suppressed or indirectly inhibited via miRNAs (or proteins associated with the synthesis of miRNAs). Inhibition of the PI3K/AKT/mTOR pathway and extracellular proteins by kaempferol prevents cell proliferation, migration, and invasion and induces death pathways such as apoptosis and autophagy. Although activities on cancer cells have been demonstrated in the scientific literature through assays of apoptosis induction and inhibition of proliferation, the molecular effect of kaempferol, a compound that may be a candidate for clinical use, requires further investigation. The impact of kaempferol on the hundreds of non-coding RNA molecules implicated in cancer should be ascertained both in vitro and in vivo, particularly by means of profiling and cloning investigations. The focus of previous research has primarily been on autophagy and apoptosis. It is further recommended to conduct experiments to evaluate the effect of kaempferol on cellular death mechanisms, particularly pyroptosis and ferroptosis.

## Figures and Tables

**Table 1:** Kaempferol and its derivatives from some plant species.

No	Compounds	Plant	References
1	kaempferol-3-O-(2-O-sinapoyl)-β-D-glucopyranosyl-(1→2)-β-D-glucopyranoside-7-O-β-D-glucopyranoside (1), kaempferol-3-O-β-D-glucopyranosyl-(1→2)-β-D-glucopyranoside-7-O-β-D-glucopyranosyl-(1→6)-β-D-glucopyranoside (2), and kaempferol-3-O-(2-O-sinapoyl)-β-D-glucopyranosyl-(1→2)-β-D-glucopyranoside-7-O-β-D-glucopyranosyl-(1→6)-β-D-glucopyranoside (3)	*Brassica juncea*	[37]
2	Kaempferol-3-O-rutinoside	*Ouratea fieldingiana*	[38]
3	Kaempferol glycosides	*Lobularia maritima*	[39]
4	Kaempferol	*Salvia hispanica*	[40]
5	Kaempferol	*Salvia brachyantha*, *S. aethiopis*, *S. microstegia*	[41]
6	Kaempferol-3-glucoside	*Rhizopus oryzae* and *R. azygosporus*	[42]
7	Kaempferol tetraglucosides	*Brassica oleracea*	[43]
8	3,7-dimethyl kaempferol ether, 7-methyl kaempferol ether	*Solanum paludosum*	[44]
9	Kaempferol, kaempferol 3-O-α-rhamnoside, kaempferol 3-O-β-glucoside	*Solanum rantonnetii*	[45]
10	Kaempferol	*Lycopersicum esculentum*	[46]
11	Kaempferol, kaempferol rhamnosides	*Hibiscus cannabinus*	[47]
12	Kaempferol	*Thymus mastichina*	[48]
13	Kaempferol-3-O-glycoside	*Hypericum triquetrifolium*	[49]
14	Kaempferol	*Teucrium polium* spp. *capitatum*	[50]
15	kaempferol-3-O-hexoside-7-O-rhamnosides, kaempferol-3-O-pentoside-4′-O-rhamnosides and kaempferol 4′,7-di-O-rhamnoside	*Prunus domestica* subsp. *syriaca*	[51]
16	Kaempferol glucoside	*Conocarpus erectus*	[52]
17	Kaempferol	*Fragaria vesca*	[53]
18	kaempferol-3,7-O-α-L-dirhamnoside, kaempferol-3-O-β-D-glucopyranosyl-(6→1)-β-L-rhamnopyranosyl-7O-α-L-rhamnopryranoside	*Bauhinia candicans*	[54]
19	Kaempferol	*Crocus sativus*	[55]
20	kaempferol-7-O-β-D-glucopyranosyl-(1→3)-β-D-glucopyranoside	*Anoectochilus roxburghii*	[56]
21	Kaempferol	*Phyllanthus maderaspatensis*	[57]

**Table 2:** Bioactivities of kaempferol on molecular pathways of various diseases.

Activity	Mechanisms	References
Anti-inflammatory	Inhibition of IL3 and IL9	[63]
Anti-diabetic	ERK phosphorylation and upregulation of vasohibin-1 level	[68]
Wound healing	Increased antioxidant activity	[2]
Anti-fibrotic	Suppression of HO-1, NOX2, IL-6, TNF-α, and TGF-β	[72]
Liver protecting	Upregulation of expression of butyrate receptors and tight junction proteins in intestinal mucosa	[73]
	Downregulation of C/EBP-homologous protein (CHOP) expression	[74]
	Upregulation of glucose-regulated/binding immunoglobulin protein 78 (Grp78) protein and suppression of ER-stress-induced apoptosis	[74]
Neuroprotective	Modulation of the GSK3-beta and Nrf2 signaling pathway	[75]
	Neuroprotective effect to reduce neuropathic pain through TLR4/NF-ĸB signaling pathway	[78]
Bone tissue healing	Regulation of Runx2 and Osx expression	[76]
Analgesic	Downregulation of myeloperoxidase, interleukin-2, TNF-alpha, and Angiopoietin-like protein 2 expression	[77]
Antioxidant activity	Upregulation of antioxidant genes HO-1, SOD1, SOD2, NQO1	[79]
Regulating activity of Multi Drug resistance	Inhibition of PKM2-mediated glycolysis	[80]
Pulmonary arterial hypertension protective activity	Inhibition of the assembly of autophagic vacuoles and lysosomes by reversing the aberrant expression of autophagy proteins such as BECN-1, LC3B-II, ATG5, ATG7, and SQSTM1	[81]

## Data Availability

No new data were created or analyzed in this study. Data sharing is not applicable to this article.
